# The Reproductive Biology and Larvae of the First Tadpole-Bearing Frog, *Limnonectes larvaepartus*

**DOI:** 10.1371/journal.pone.0116154

**Published:** 2015-01-02

**Authors:** Mirza D. Kusrini, Jodi J. L. Rowley, Luna R. Khairunnisa, Glenn M. Shea, Ronald Altig

**Affiliations:** 1 Department of Forest Resources Conservation and Ecotourism, Bogor Agricultural University, Bogor, Indonesia; 2 Australian Museum Research Institute, Australian Museum, Sydney, NSW, Australia; 3 Faculty of Veterinary Science, University of Sydney, Sydney, NSW, Australia; 4 Department of Biological Sciences, Mississippi State University, Mississippi State, MS, United States of America; Leibniz-Institute of Freshwater Ecology and Inland Fisheries, Germany

## Abstract

Most of the reproductive modes of frogs include an exotrophic tadpole, but a number of taxa have some form of endotrophic development that lacks a feeding tadpole stage. The dicroglossid frog genus *Limnonectes* ranges from China south into Indonesia. The breeding biologies of the approximately 60 described species display an unusual diversity that range from exotrophic tadpoles to endotrophic development in terrestrial nests. There have been mentions of oviductal production of typical, exotrophic tadpoles in an undescribed species of *Limnonectes* from Sulawesi, Indonesia. Here we examine newly collected specimens of this species, now described as *L. larvaepartus* and present the first substantial report on this unique breeding mode. Typical exotrophic tadpoles that are retained to an advanced developmental stage in the oviducts of a female frog are birthed into slow-flowing streams or small, non-flowing pools adjacent to the streams.

## Introduction

Although most reproductive modes of frogs include an exotrophic, or externally feeding tadpole [1], a sizeable number of frogs have some form of endotrophic development. These embryos obtain their developmental energy from parental sources (i.e., usually egg yolk) and a feeding tadpole stage is absent. Fertilization is typically external in frogs, and the few cases of internal fertilization are accomplished in several different ways. Internal fertilization does not always result in births; *Eleutherodactylus coqui*, and perhaps many other confamilial taxa, has internal fertilization [2] but deposits terrestrial, direct-developing eggs soon after amplexus is completed.

The approximately 60 known species of the dicroglossid genus *Limnonectes* range from China south into Indonesia [3]. This genus includes an unusual diversity of breeding biologies that range from exotrophic tadpoles to endotrophic development in terrestrial nests [4], and there have been mentions of an undescribed species of *Limnonectes* (*Limnonectes* sp. V [5]) having the unique oviductal production of tadpoles that exhibit the morphology of typical exotrophic tadpoles [6]–[8]. This species is now formally described as *L. larvaepartus* by Iskandar *et al.* [9]. We collected specimens of this species and describe the unique breeding mode and the larva. Typical exotrophic tadpoles are retained to an advanced developmental stage in the oviducts of female frogs and are birthed into slow-flowing streams.

## Materials and Methods

### Ethics statement

Permission to conduct the survey, including specimen collection, was granted from the North Sulawesi Forestry Office (BKSDA Sulawesi Utara) in Manado under the name of L. R. Khairunnisa (permit number 23/SIMAKSI/BKSDA-SU/2013) and M. D. Kusrini (permit number 07/SATS-DN/BKSDA.SU/PKHM/2014). Protocols for surveying and specimen handling were approved by the Ministry of Education. Specimen collection was approved by LIPI (Lembaga Ilmu pengetahuan Indonesia; the Indonesian Institute of Science) as part of obtaining field permits.

### Specimen collection

**Figure 1:**
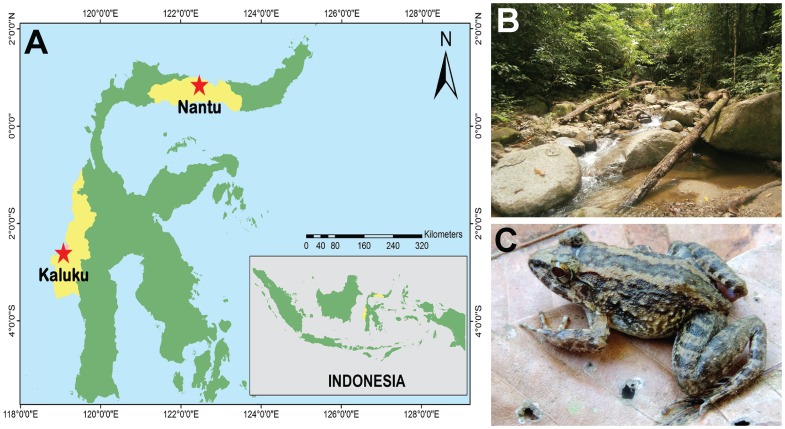
Collection locality, habitat and live *Limnonectes larvaepartus.* (A) Collection locality of *Limnonectes larvaepartus* at Bontula, Nantu Wildlife Sanctuary, Gorontalo Province, Indonesia, (B) habitat of *L. larvaepartus* at Bontula, and (C) an adult female *L. larvaepartus* (MZB Amp 23978) in life.

Frogs and tadpoles of *Limnonectes larvaepartus* were collected at Bontula, Nantu Wildlife Sanctuary, Gorontalo Province, Indonesia (0°50′51″N, 122°28′19″E; Fig. 1A, 1B). Frogs were captured by hand and tadpoles by dip net. Frogs and tadpoles were anesthetized using Tricaine methane sulfonate (MS222). Adult specimens were preserved and stored in 70% ethanol and samples of thigh muscle tissue were preserved in 100% ethanol for molecular analysis. All *L. larvaepartus* specimens were deposited at the Museum Zoologicum Bogoriense (MZB).

We examined five adult *Limnonectes larvaepartus* in detail: a gravid female (MZB Amp 22973), a non-gravid female (MZB Amp 23259), and a male (MZB Amp 23265) collected on 27 June 2013, and two gravid females (MZB Amp 23978; Fig. 1C and MZB Amp 23979) collected on 26 May and 24 May 2014 respectively. All adult *L. larvaepartus* were caught along a sandy-bottomed stream (Fig. 1B) at the border between a village and the forest.

*Limnonectes larvaepartus* tadpoles were collected in the same stream as the adults. Specimens collected on 12 June 2014 (MZB Amp 23980) were found in a small pool (24 cm deep) with a substrate of sand and leaf litter within the stream and others (MZB Amp 23982) were collected on 26 May 2014 in a rock pool (28 cm deep) disconnected from the main stream. Two tadpoles from MZB Amp 23980 and two from MZB Amp 23982 were examined in detail.

### Specimen examination

Gravid *Limnonectes larvaepartus* were dissected to determine the number, stages, and morphology of the tadpoles and the relative characteristics of the ovaries and fat bodies as indicators of their reproductive cycle. The tadpoles were described in the contexts of Altig and McDiarmid [10] and the staging table of Gosner [11]. Measurements of tadpole were made with calipers or an ocular micrometer (0.1 mm) as follows: total length (TL) from tip of snout to tip of tail; tail length (TAL) from the body terminus to the tip of the tail; body length (BL) from tip of the snout to the body terminus; maximum tail height (MTH) as the greatest vertical height of the tail; tail muscle height (TMH) at the base, tail muscle width (TMW) at the base; and interorbital distance (IOD) measured between centers of the pupils.

Standard histological sections (alcohol dehydration series, embedded in paraffin, sections cut at 8 µm and stained with Hemotoxylin and Eosin) of the oviducts of one gravid (MZB Amp 22973) and non-gravid female (MZB Amp 23259) were made to evaluate if any oviductal modifications were present. The characteristics of the testes and fat bodies of a syntopic male were also recorded.

### Molecular analysis

We analyzed samples from three frogs (MZB Amp 22973, 23259, 23265) and all four tadpoles examined from Nantu to confirm their identity. Total genomic DNA was extracted with DNeasy tissue extraction kits (Qiagen), and we used the primers 16SAR and 16SBR of Palumbi et al. [12] to amplify ∼500 base pairs of the 16S rRNA gene. Standard PC protocols were used and PCR products were purified with ExoSap-IT (USB Corporation, OH, USA). Purified templates were sequenced directly by Macrogen (Seoul, Korea). Sequences were validated with Sequencher 4.10 (Gene Codes, Ann Arbor, MI), aligned with the Clustal option in MEGA-5, and refined by eye. Uncorrected pairwise sequence divergence was calculated with MEGA-5. Sequences were deposited in GenBank under the accession numbers KM655288−KM65529.

## Results

The identities of Nantu specimens were confirmed molecularly as *Limnonectes larvaepartus* (0.2−0.4% sequence divergence at the 16S rRNA gene fragment from *Limnonectes* sp. V; GenBank accession number JF744609).

### Reproductive Mode

**Figure 2:**
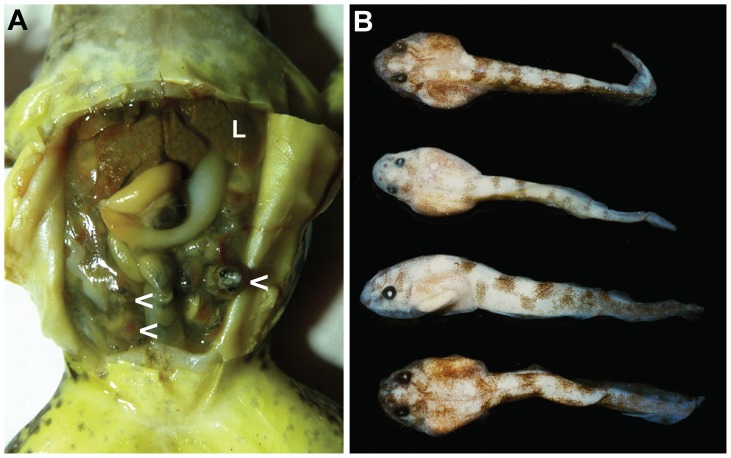
Gravid *Limnonectes larvaepartus* with oviductal tadpoles. (A) Intact viscera of gravid female (MZB Amp 22973; L = liver and arrows = several tadpoles), and (B) four of the preserved tadpoles removed from the oviducts of the same female.

In life, movements of the sides of the abdomen of the gravid female (MZB Amp 22973, 55.7 mm SVL; Fig. 2A) were observed. When this female was dissected, 46 tadpoles in stage 25 were found in the left oviduct and 50 were in the right oviduct; clutch size at the time of capture was 103 (i.e. 46+50+7 tadpoles ejected upon capture). The tadpoles measured 13.4−14.0 mm TL (Fig. 2B). These tadpoles were tightly packed haphazardly in the greatly enlarged, very thin-walled, and translucent oviduct (about 1.0 cm diameter) and filled all the space in her abdomen. There was no debris suggestive of egg membranes, dead tadpoles, or feces in the oviducts. Some tadpoles had some yolk reserves in the anterior part of the gut and all had brown, particulate material in the posterior three-quarters of their intestines. The dense fat bodies of the females were dull yellow and about 8 mm long with four projections from a common base; individual adipocytes were not visible. The middle and distal part of the intestine contained remnants of food items. The ovaries of MZB Amp 22973 were pushed anteriorly into a globular shape near the base of the liver by the volume of the tadpoles in the oviducts.

Tadpoles had already been ejected from the vent of the other gravid females: 42 from MZB Amp 23978 and 18 from MZB Amp 23979. Neither of these females had tadpoles remaining in the oviducts. Their ovaries were less compressed.

The ovaries of all gravid females contained a few white, immature oocytes (0.4−0.7 mm diameter) and more numerous and larger (1.6−1.8 mm diameter), pigmented ova. The non-gravid female (MZB Amp 23259, 48.1 mm SVL) had greatly expanded ovaries with large, pigmented oocytes (about 1.8−2.0 mm diameter) arranged almost in a monolayer across the roof of the abdominal cavity) and a few small white oocytes. The ovaries were in a more normal position and shape relative to those described above. The testes of a male (MZB Amp 23265; 54.0 mm SVL; 4.4×3.1 mm) were pale yellow.

Histological sections of the very thin-walled oviduct of one gravid female (MZB Amp 22973) showed no obvious specializations. The oviductal epithelium was simple cuboidal to low columnar with no obvious aggregations of secretory cells or any surface ornamentation. The lamina propria was well vascularised, the submucosa contained scattered larger arteries and veins, and the lamina propria and submucosa lacked glands. The tunica muscularis was thin, and in some places the smooth muscle fibres were widely spread apart. The oviducts of the two other gravid females (MZB Amp 23978−23979) were preserved within 24 h of shedding the last tadpoles, and the structures had begun to involute with the oviductal wall grossly thicker than in MZB Amp 22973. The oviduct were still significantly dilated throughout their length and filled much of the coelom. The oviduct of the non-gravid female was densely white, straight for the anterior half and contorted in the distal half.

### Tadpoles

**Figure 3:**
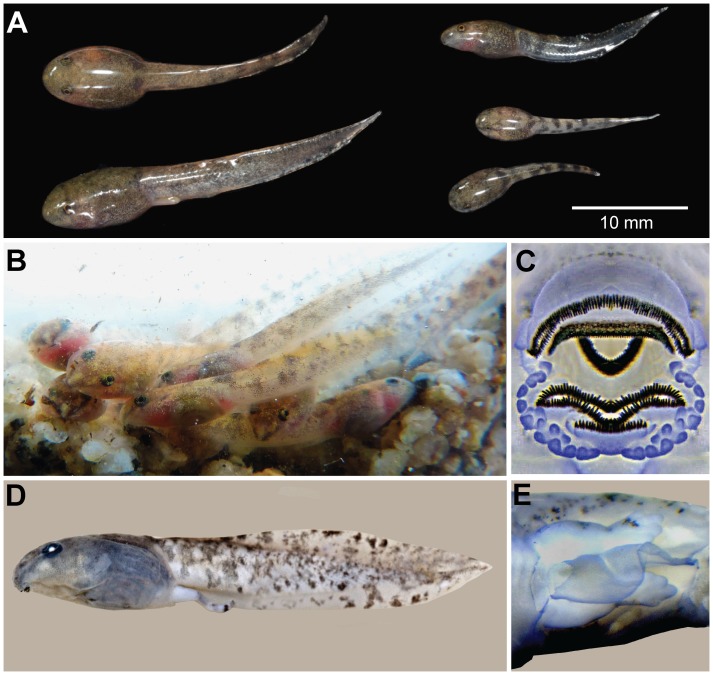
Free swimming *Limnonectes larvaepartus* tadpoles. (A) Tadpoles from Kaluku, West Sulawesi (JAM 14268; stages 25, 29 and 34–36) collected from a small pool at the margin of a stream, (B) tadpoles ejected from MZB Amp 23978 at stages 26–27, (C) oral disc of MZB Amp 23982a at stage 31, (D) lateral view of MZB Amp 23980a at stage 36, and (E) the vent tube of MZB Amp 23980b at stage 31.

The oviductal tadpoles that we examined were 13.4−14.0 mm TL and had a labial tooth row formula of 1/2 at stage 25 (Fig. 2A, 2B). Free swimming tadpoles found in Nantu were from Gosner stage 31−34 with a total length of 28.2 mm (stage 31) to 29.4 mm (stage 34; Table 1). Free swimming tadpole confirmed molecularly as *L. larvaepartus* were also found in Kaluku, West Sulawesi in July 2014. These tadpoles were in Gosner stages 25−37 (J. McGuire, personal communication) at a total length of 14.3 mm (stage 25) to 29.4 mm (stage 36; Fig. 3A). Tadpoles at stage 25 ejected from a female from Nantu (MZB Amp 23978; Fig. 3B) lived in an aquarium for two weeks without additional food.

**Table 1:** Gosner (1960) stage and measurements (mm) of four *Limnonectes larvaepartus* tadpoles from Nantu.

	MZB Amp 23982a	MZB Amp 23980b	MZB Amp 23982b	MZB Amp 23980a
**Gosner Stage**	31	31	34	36
**TL**	29.1	–	28.2	29.4
**TAL**	20.0	–	19.0	19.7
**BL**	8.7	8.7	8.9	9.2
**MTH**	3.5	4.5	3.5	4.9
**TMH**	2.8	3.3	2.9	3.3
**TMW**	2.6	2.1	2.4	2.8
**IOD**	2.6	3.1	2.8	3.3

See methods for abbreviations. MZB Amp 23980b had a damaged tail tip.

A detailed tadpole description is based upon four *L. larvaepartus* tadpoles (MZB Amp 23980a, b; 23982a, b) in Gosner stages 31−36 (Table 1; Fig. 3C−E). The sinistral spiracle is about midway between the eye and vent, opens posterodorsally and has a definite medial wall. The dextral vent tube (Fig. 3E, Amp 23980b) has a large bore that widens distally, and the right hand wall inserts well anterior to the left wall of the tube. In this preserved specimen, the body is indistinctly mottled with punctate melanophores that are absent on the spiracle, and the venter is clear enough that the intestine and some of the jaw musculature can be seen. The eyes are distinctly dorsal.

The tail muscle and fins are randomly marked with small, dark blotches on a pale background throughout (Fig. 3D); dark bands on the tail muscle that occur in some species of *Limnonectes* are not well defined.

The oral disc faces almost ventrally, and the labial tooth row formula is 2(2)/3 with a wide gap in the second upper row (Fig. 3C, MZB Amp 23982a). The rather large labial teeth are coarsely spaced. The narrow upper jaw sheath with fine serrations forms a wide arc, and the lower sheath is openly U-shaped. The uniserial marginal papillae end near the lateral ends of the first upper tooth row, and there is a wide dorsal and a narrow ventral gap. The short, third lower row sits within the gap in the marginal papillae, and four submarginal papillae extend laterally from the ends of the third lower tooth row.

## Discussion

Production of typical exotrophic tadpoles in the oviducts of a frog is a reproductive novelty. There are cases in which a froglet is released from the oviducts (e.g., one *Eleutherodactylus* and *Nimbaphrynoides*), and tadpoles or froglets may be birthed from other parts of a parent’s body (e.g., *Assa, Gastrotheca, Rheobatrachus* and *Rhinoderma*), but no other species is known to give birth to typical tadpoles. All available information suggests that female *Limnonectes larvaepartus* releases their young as typical exotrophic tadpoles in slow parts of streams similar to where the gravid female was found. The larvae do not have sufficient yolk reserves to carry them through metamorphosis and there was no evidence that nutrients are supplied by the oviductal wall. Embryos in the oviducts in some African bufonids are provided with nutritional uterine secretions after the yolk is exhausted, and some indication of nutrient transfer is suspected in *Rhinoderma* [13]. Cycles of fat storage in tropical frogs are poorly studied, but the fat bodies of the gravid female examined were not depleted. This, and the presence of food material in the females’ intestine, suggests that the females are not energetically stressed by carrying oviductal young.

Many questions addressing the eggs, embryos and the female reproductive tract need to be answered before we will fully understand this new breeding mode. Perhaps the most intriguing question asks if embryos of any exotrophic frog could develop in the oviduct if sperm were present. The functional plasticity of the oviduct may be generally amenable to embryonic development but the behavior to fertilize the eggs is not present. Internal fertilization by cloacal apposition seems like a relatively minor behavioral change from typical, amplectic external fertilization [2], although the mode of fertilization does not always predict further reproductive processes.

The oviductal tadpoles of *L. larvaepartus* digest vitellogenic yolk until that is depleted, and dark, particulate material in the gut of advanced tadpoles suggest that feces or dead individuals are eaten; cannibalism and scavaging is common among tadpoles and should not be discounted. Larval transport on a parent’s back is known in *Limnonectes* from Borneo [14], [15], but these males carried tadpoles that probably developed in a terrestrial nest. The transported tadpoles, some in the process of being released in non-flowing water, were considerably smaller and less developed than the tadpoles from the oviducts of *L. larvaepartus*. Dissections of museum specimens and field observations are needed in order to shed light on this strange breeding mode.

Evolutionary hypotheses on reproductive transitions and the origin of giving birth to tadpoles in *Limnonectes* is not possible with the available data. The true species diversity in the genus is unknown [16], and knowledge of the reproductive biology is absent in most cases [5], [17]. Further examination of related species may reveal additional species in the genus that display similar reproductive biology to *L. larvaepartus*, particularly in the closely related *L. heinrichi* [5].

## References

[1] Haddad CFB, Prado CPA (2005) Reproductive modes in frogs and their unexpected diversity in the Atlantic Forest of Brazil. BioScience 55:207–217.

[2] Townsend DS, Stewart MM, Pough FH, Brussard PF (1981) Internal fertilization in an oviparous frog. Science 212:469–471. 6894203 10.1126/science.6894203

[3] Frost DR (2014) *Amphibian Species of the World: an online reference. Version 6.0.* (research.amnh.org/vz/herpetology/amphibia/). American Museum of Natural History, New York, USA.

[4] Rowley JJL, Altig R (2012) Nidicolous development in *Limnonectes limborgi* (Anura: Dicroglossidae). Amphib-Reptil 33:145–149.

[5] Setiadi MI, McGuire JA, Brown RM, Zubairi M, Iskandar DT, et al (2011) Adaptive radiation and ecological opportunity in Sulawesi and Philippine fanged frog (*Limnonectes*) communities. Amer Nat 178:221–240. 21750386 10.1086/660830

[6] Brown RM (2009) Frogs in island archipelagos. In: Gillespie R, Clague D, eds. Encyclopedia of Islands, Berkeley: University of California Press. 347–351.

[7] Iskandar DT, Erdelen WR (2006) Conservation of amphibians and reptiles in Indonesia: issues and problems. Amphib Reptil Conserv 4:60–87.

[8] Iskandar DT, Tjan KN (1985) The amphibians and reptiles of Sulawesi, with notes on the distribution and chromosomal number of frogs. In: Kitchener DJ, Suyanto A, eds. Proceedings of the First International Conference on Eastern Indonesian-Australian Vertebrate Fauna. Manado, Indonesia. 39–45.

[9] Iskandar DT, Evans BJ, McGuire JA (2014) A novel reproductive mode in frogs: A new species of frog with internal fertilization and birth of tadpoles. PLoSOnexxxx. 10.1371/journal.pone.0115884 PMC4281041 25551466

[10] Altig R, McDiarmid RW (1999) Body plan: development and morphology. In: McDiarmid RW, Altig R, eds. Tadpoles: the biology of anuran larvae. Chicago: University of Chicago Press. 24–51.

[11] Gosner KL (1960) A simplified table for staging anuran embryos and larvae with notes on identification. Herpetologica 16:183–190.

[12] Palumbi SR, Martin A, Romano S, McMillan WO, Stice L, et al**.** (1991) The simple fool’s guide to PCR. Honolulu: Department of Zoology, University of Hawaii. 47 p.

[13] Goicoechea O, Garrido O, Jorquera B (1986) Evidence for a trophic relationship in the frog *Rhinoderma darwinii* . J Herpetol 20:168–178.

[14] Inger RF, Voris HK, Walker P (1986) Larval transport in a Bornean ranid frog. Copeia 1986:523–525.

[15] Inger RF, Voris HK (1988) Taxonomic status and reproductive biology of Bornean tadpole-carrying frogs. Copeia 1988:1060–1062.

[16] McLeod DS (2010) Of Least Concern? Systematics of a cryptic species complex: *Limnonectes kuhlii* (Amphibia: Anura: Dicroglossidae). Mol Phylogenet Evol 56:991–1000. 20385247 10.1016/j.ympev.2010.04.004

[17] Evans BJ, Brown RM, McGuire JA, Supriatna J, Andayani N, et al (2003) Phylogenetics of fanged frogs: testing biogeographical hypotheses at the interface of the Asian and Australian faunal zones. Syst Biol 52:794–819. 14668118

